# Identification of a Novel Bacterial Outer Membrane Interleukin-1Β-Binding Protein from *Aggregatibacter* *actinomycetemcomitans*

**DOI:** 10.1371/journal.pone.0070509

**Published:** 2013-07-31

**Authors:** Annamari Paino, Tuuli Ahlstrand, Jari Nuutila, Indre Navickaite, Maria Lahti, Heidi Tuominen, Hannamari Välimaa, Urpo Lamminmäki, Marja T. Pöllänen, Riikka Ihalin

**Affiliations:** 1 Department of Biochemistry and Food Chemistry, University of Turku, Turku, Finland; 2 Haartman Institute, Department of Virology, University of Helsinki, Helsinki, Finland; 3 Helsinki University Hospital Laboratory (HUSLAB), Helsinki University Hospital, Helsinki, Finland; 4 Institute of Dentistry, University of Turku, Turku, Finland; Fundación Investigación Sanitaria Illes Balears, Spain

## Abstract

*Aggregatibacter* *actinomycetemcomitans* is a gram-negative opportunistic oral pathogen. It is frequently associated with subgingival biofilms of both chronic and aggressive periodontitis, and the diseased sites of the periodontium exhibit increased levels of the proinflammatory mediator interleukin (IL)-1β. Some bacterial species can alter their physiological properties as a result of sensing IL-1β. We have recently shown that this cytokine localizes to the cytoplasm of *A. actinomycetemcomitans* in co-cultures with organotypic gingival mucosa. However, current knowledge about the mechanism underlying bacterial IL-1β sensing is still limited. In this study, we characterized the interaction of *A. actinomycetemcomitans* total membrane protein with IL-1β through electrophoretic mobility shift assays. The interacting protein, which we have designated bacterial interleukin receptor I (BilRI), was identified through mass spectrometry and was found to be *Pasteurellaceae* specific. Based on the results obtained using protein function prediction tools, this protein localizes to the outer membrane and contains a typical lipoprotein signal sequence. All six tested biofilm cultures of clinical *A. actinomycetemcomitans* strains expressed the protein according to phage display-derived antibody detection. Moreover, proteinase K treatment of whole *A. actinomycetemcomitans* cells eliminated BilRI forms that were outer membrane specific, as determined through immunoblotting. The protein was overexpressed in *Escherichia coli* in both the outer membrane-associated form and a soluble cytoplasmic form. When assessed using flow cytometry, the BilRI-overexpressing *E. coli* cells were observed to bind 2.5 times more biotinylated-IL-1β than the control cells, as detected with avidin-FITC. Overexpression of BilRI did not cause binding of a biotinylated negative control protein. In a microplate assay, soluble BilRI bound to IL-1β, but this binding was not specific, as a control protein for IL-1β also interacted with BilRI. Our findings suggest that *A. actinomycetemcomitans* expresses an IL-1β-binding surface-exposed lipoprotein that may be part of the bacterial IL-1β-sensing system.

## Introduction

*Aggregatibacter* *actinomycetemcomitans* is a Gram-negative opportunistic human pathogen that causes chronic and aggressive forms of periodontitis [1-3]. This pathogen is also associated with systemic diseases, such as cardiovascular diseases [4,5], and it possesses variety of virulence properties (reviewed in 6), which enhances its resistance against human defense mechanisms.

*A. actinomycetemcomitans* coaggregates with other oral species [7,8] and forms robust biofilms [9,10], which may partly explain the high tolerance of the species to host clearance mechanisms and antibiotics [11,12]. Biofilm cells generally show a different pattern of gene expression than their planktonic counterparts. For example, an important periodontal pathogen, *Porphyromonas gingivalis*, exhibits expression of stress response genes following quorum-sensing events, contributing to pathogen survival during infection [13,14]. In a multispecies biofilm model, the presence of the periodontal pathogens *A* *. actinomyctemcomitans* and *P. gingivalis* has been shown to cause changes in the gene expression profiles of the commensal strains in the biofilm [15]. *A. actinomycetemcomitans* employs several specific strategies for enhancing its survival in the host: it can secrete a leukotoxin that directly targets human phagocyte cells, monocytes and macrophages [16-18], while the other toxin produced by the species, cytolethal-distending toxin, indirectly modulates periodontal bone resorption [19] as well as periodontal keratinocyte and fibroblast proliferation [20,21]. However, little is known about the ability of the species to adjust its virulence properties after sensing inflammation-related environmental factors.

High levels of the human proinflammatory mediator interleukin (IL)-1β are typical of periodontal inflammation sites in tooth-supporting tissues [22]. IL-1β is a key inflammatory mediator of the human innate immune system. This cytokine is secreted primarily by human macrophages and monocytes after sensing microbial danger signals. IL-1β stimulates its own secretion [23], and the bursts of IL-1β that are released from human cells are highly controlled (reviewed in 24). Human mononuclear leukocytes detect *A. actinomycetemcomitans* as a pathogen via NLRP3 inflammasome [25], which induces the maturation of pro-IL-1β to its biologically active form. Dysregulation of IL-1β activity can result in chronic diseases such as periodontitis (reviewed in 24,26). *In vitro*, virulent bacteria may sense the inflammatory levels of this signal by binding it and altering their growth properties [27-29]. Furthermore, IL-1β is known to modulate virulence gene expression in *Staphylococcus aureus* [30]. In previous studies, we observed that *A. actinomycetemcomitans* biofilm sequestered and then took up human IL-1β [31]. In addition, *A. actinomycetemcomitans* increases the biofilm mass as a physiological response to IL-1β [32], similar to the biofilms formed by Gram-positive *S. aureus* [28,29]. As a secondary response, IL-1β binding temporarily decreases the metabolic activity of this species [32].

The findings of the studies described above strongly suggest that bacteria may use IL-1β as an indicator of the host inflammatory state. However, the mechanisms underlying the uptake and subsequent regulatory pathway of IL-1β in bacteria are not known. Currently, the only bacterial IL-1β-binding outer membrane receptor has been characterized from the Gram-negative bacterium *Yersinia pestis* [33]. Gram-negative *Pseudomonas aeruginosa* has been shown to specifically sense interferon (IFN)-γ via its outer membrane protein OprF [34]. The interleukin receptor of *Yersinia* is known as capsule antigen F1 assembly (Caf1A) protein. Caf1A is required for the outer membrane localization of capsule antigen F1 protein, which shows significant sequence similarity to a human IL-1α, β receptor antagonist [35]. According to BLAST searches, the *A. actinomycetemcomitans* genome does not show significant sequence similarity with genes encoding Caf1A, Caf1 or the human interleukin-1 receptor. Thus, we sought to specifically study the mechanism of IL-1β transfer inside bacterial cells using the oral pathogen *A. actinomycetemcomitans* as a model species.

In this paper, we describe the interaction of human IL-1β with a hypothetical lipoprotein extracted from *A. actinomycetemcomitans* from a dissolved total membrane protein fraction. Based on the observed interaction, we designated this putative protein bacterial interleukin-1β receptor I (BilRI). The interacting protein was identified via mass spectrometry (MS) analysis, and its subcellular location was analyzed using subcellular localization prediction tools for bacterial proteins. The putative bacterial outer membrane IL-1β receptor was overexpressed in an *Escherichia coli* strain designed for membrane protein overexpression [36] and was also produced in a soluble form in *E. coli*, without the typical lipoprotein signal sequence. The *E. coli* cells containing BilRI in their outer membrane showed an increased IL-1β-binding capacity, and the soluble form of the protein interacted with IL-1β in a microplate assay. Additionally, all of the tested clinical *A. actinomycetemcomitans* strains expressed BilRI, and the localization of the protein in the outer membrane was verified through proteinase K treatment. Knowledge of bacterial mechanisms for the binding and uptake of the central inflammatory cytokine IL-1β is essential for understanding the behavior and properties of the opportunistic pathogen under inflammatory conditions.

## Results

### Identification of an IL-1β-binding membrane protein in *A. actinomycetemcomitans*

**Figure 1:**
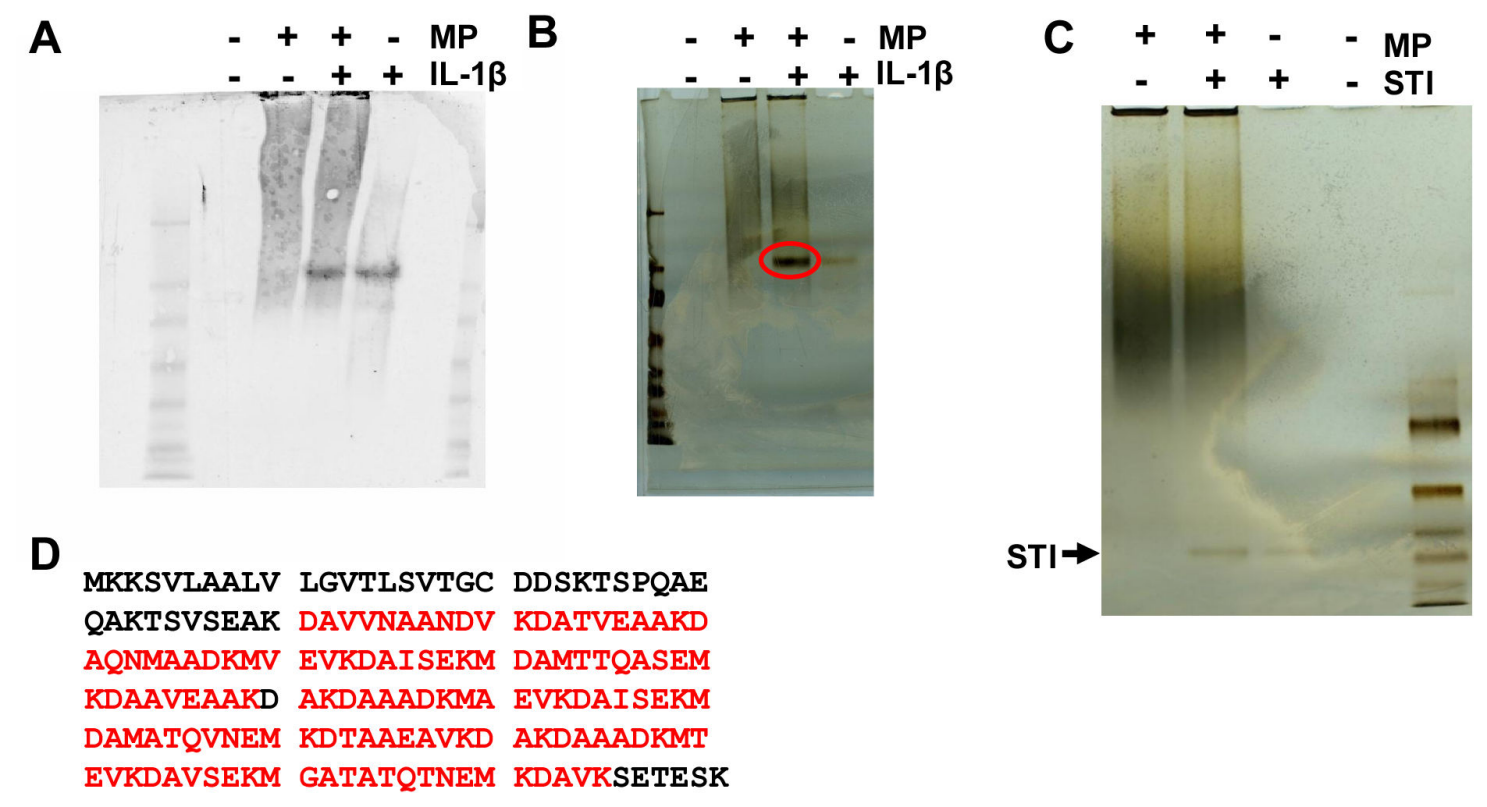
The uncharacterized putative membrane protein from *A. actinomycetemcomitans* interacted with IL-1β. Membrane proteins (MP) were isolated from *A. actinomycetemcomitans* and dissolved in 4% CHAPS, after which they were incubated with recombinant human IL-1β. The samples were subjected to native-PAGE and either blotted with anti-IL-1β (A) or silver stained (B). Anti-IL-1β detected similar amounts of IL-1β in samples containing MP+IL-1β and a sample of pure IL-1β (A). In the silver-stained gel, the equivalent band was more intense in the MP+IL-1β sample (B; red circle) than in the IL-1β sample, indicating the presence of proteins other than IL-1β. This phenomenon was not observed in the control experiment using the soybean trypsin inhibitor (STI) protein as a similar size control (C). The intense protein band (B; red circle) was cut out of the silver-stained gel and identified as the putative uncharacterized G3ZCI1 protein from *A. actinomycetemcomitans* D17P-3 via LC-MS/MS analysis. The identified peptides (D; bold red) covered 72% of the total protein sequence.

The protein band that interacted with the anti-IL-1β antibody was only visible in lanes containing either IL-1β or the IL-1β-membrane protein (MP) combination (Figure 1A). The intensity of this band was the same in both of these lanes (Figure 1A). However, in a silver-stained gel, the band was much more intense in the lane that contained both IL-1β and MP than in the lane containing only IL-1β (Figure 1B). The band was missing from the lane containing pure MP (Figure 1B). This phenomenon was not observed when IL-1β was replaced with the control protein soybean trypsin inhibitor (STI) (Figure 1C). Thus, it was suggested that in the IL-1β-MP lane, the intense band also contained proteins other than IL-1β and that those proteins most likely interacted with IL-1β. The intense protein band was extracted following non-denaturing-PAGE and identified as putative uncharacterized protein G3ZCI1 of *A. actinomycetemcomitans* D17P-3 through LC-MS/MS analysis. The identified peptides covered 72% of the total protein sequence (Figure 1D). In addition to *A. actinomycetemcomitans* protein G3ZCI1, IL-1β was detected in the band, confirming the potential interaction of the novel protein with IL-1β. As the bacterial protein had no previously known function, we designated it bacterial interleukin receptor I (BilRI).

**Figure 2:**
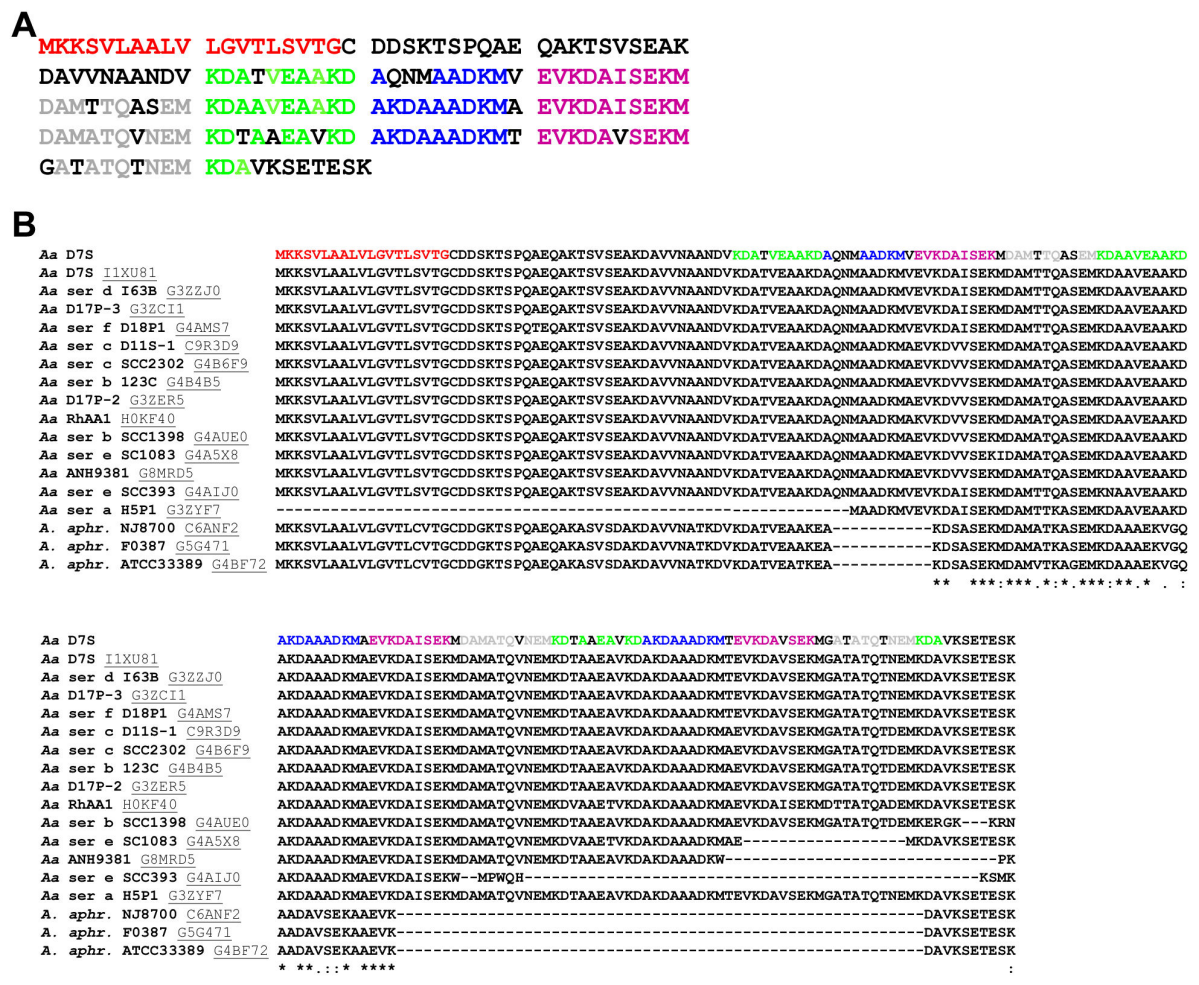
The bacterial IL-1β-binding protein consisted of a lipoprotein signal sequence and four repeated sequences. Sequence analysis using the SignalP 4.1 Server [54] revealed that the bacterial IL-1β-binding protein, which we designated bacterial interleukin receptor I (BilRI), contained a putative signal sequence of 19 amino acids (A and B; bold red). The sequence also contained 4 different 10-amino acid-long sequences (A and B; bold green, blue, purple and grey) repeated in the same order three times. BLAST similarity searches [56] and Clustal W sequence alignment [57] revealed almost identical sequences in other *A. actinomycetemcomitans* (*Aa*) strains and *A* *. aphrophilus* ( *A* *. aphr* *.*) (B).

**Figure 3:**
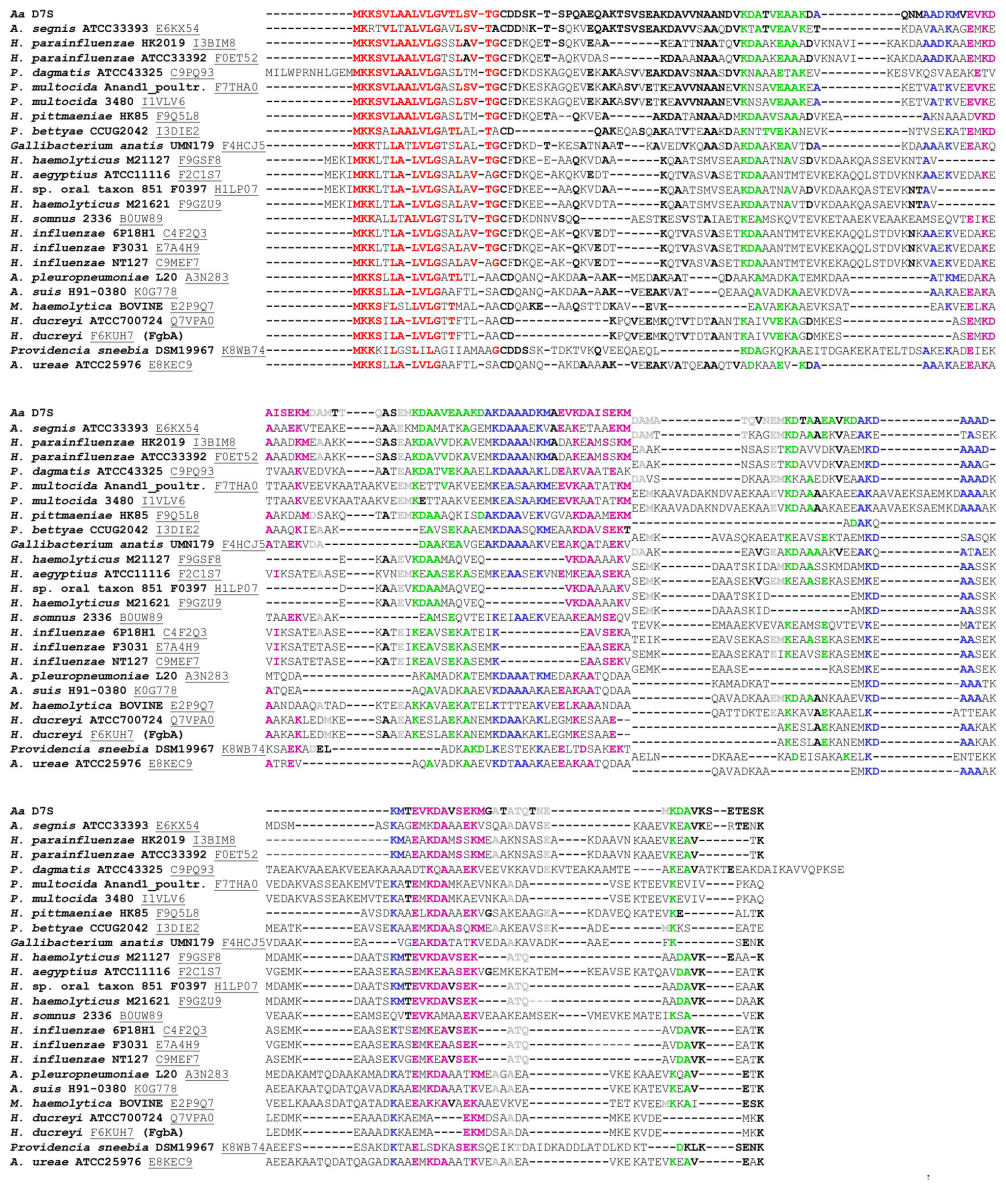
Only *Pasteurellaceae* species possessed proteins showing sequence similarity to BilRI. BLAST similarity searches [56] and Clustal W sequence alignment [57] only detected similar proteins in species belonging to the *Pasteurellaceae* family. The proteins with the highest similarity were selected for further analysis. A bolded font indicates a similar amino acid to that seen in BilRI. Different colors were used to distinguish the signal sequence (red) and the repeating sequences (green, blue, purple and grey) from the other parts of the sequence (black).

The amino acid sequence of BilRI was subjected to a BLAST search against the genome sequence of *A. actinomycetemcomitans* D7S, and an identical protein was found. The BilRI sequence contained three almost identical repeating sequences, which covered approximately 75% of the mature protein sequence (Figure 2A). A similar protein was found in several *Pasteurellaceae* species (Figures 2B and 3), but not in *E. coli*. Its first 19 amino acids likely form the lipoprotein signal sequence, which directs the protein to the outer membrane in Gram-negative species (Figure 2B).

### Recombinant BilRI localized to the outer membrane of *E. coli*

**Figure 4:**
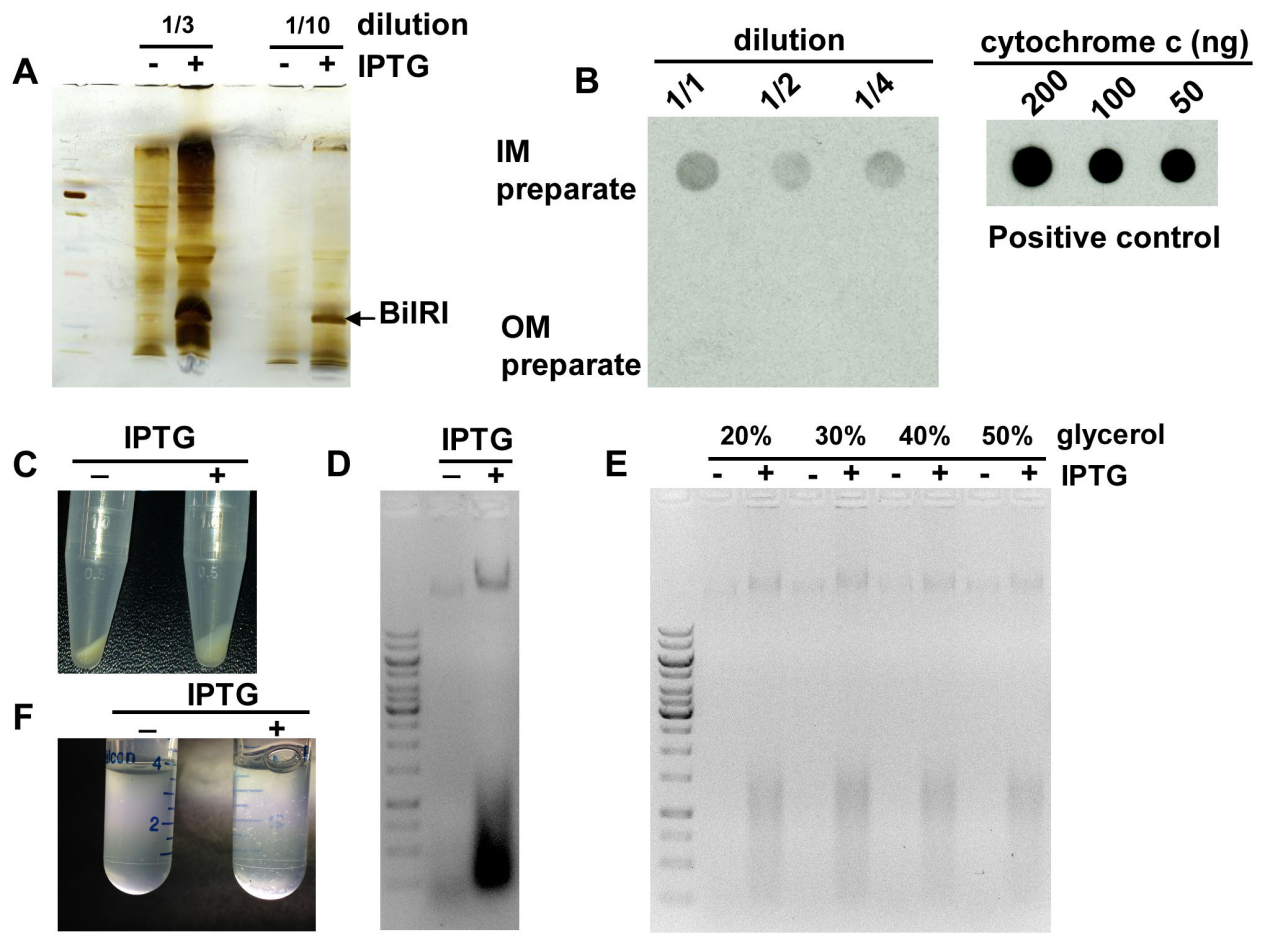
Recombinant *E. coli* expressing BilRI in the outer membrane showed membrane frailty and self-aggregation. The *A. actinomycetemcomitans bilRI* gene, including the signal sequence, was cloned into *E. coli*. The production of BilRI was induced using IPTG. The outer membrane fraction was isolated from both induced (IPTG+) and non-induced (IPTG-) cells, and different dilutions of the membrane samples were subjected to SDS-PAGE, followed by silver staining (A). The purity of the outer membrane (OM) fraction was confirmed by checking for the presence of inner membrane (IM) heme proteins using ECL [59]. Only the IM preparate and positive control cytochrome c generated a signal indicating the presence of heme (B). When IPTG-induced recombinant *E. coli* cells were frozen in the absence of protective agents, the cells began to break down, which was observed in the form of a spongy bacterial pellet (C) and high levels of DNA released in the cell supernatant (D). The presence of glycerol during freezing prevented the cells from breakdown, as they released less DNA during resuspension (E). When the IPTG-induced *E. coli* cells were incubated in the presence of 50 mM CaCl_2_ at 37^°^C for 1 hour, they started to self-aggregate (F).

When BilRI was expressed in *E. coli* with its native signal sequence, the outer membrane protein fraction contained the recombinant protein (Figure 4A), which was identified from a silver-stained gel via LC-MS/MS. The purity of the outer membrane protein fraction was confirmed through the detection of heme proteins, which are only present in the inner membrane fraction [37] (Figure 4B).

### Overexpression of BilRI in the *E. coli* outer membrane increased membrane frailty and caused self-aggregation in the presence of calcium

The production of large amounts of recombinant BilRI in the outer membrane of *E. coli* led to increased frailty of *E. coli* cells (Figure 4C–E). When a bacterial pellet was frozen at -20^°^C and resuspended in PBS, the suspension was more viscous when it contained IPTG-induced cells than non-induced cells. Additionally, after centrifugation, the pellet was looser (Figure 4C), and the supernatant contained a large amount of DNA (Figure 4D), indicating that the outer membrane BilRI-producing *E. coli* cells broke down during the freezing and thawing cycle. However, 50% glycerol protected the cells from breakdown (Figure 4E).

When the *E. coli* cells producing native BilRI were incubated in the presence of 50 mM calcium, they began to aggregate. This phenomenon was not observed in non-induced *E. coli* cells (Figure 4F).

### Recombinant BilRI increased the binding of IL-1β to *E. coli*

**Figure 5:**
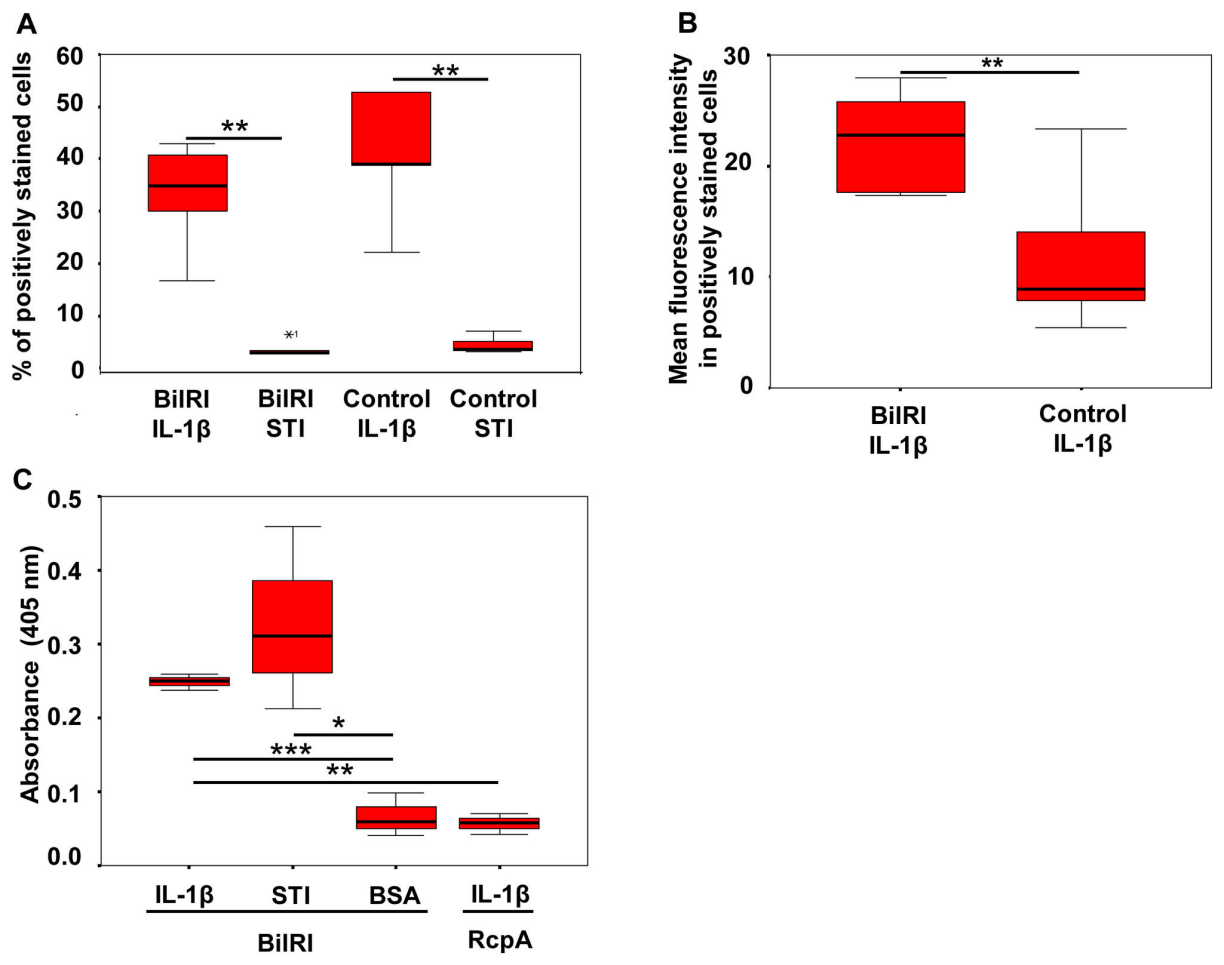
Recombinant BilRI bound IL-1β both in the outer membrane of *E. coli* and as soluble protein. The IL-1β-binding capacity of recombinant *E. coli* cells containing BilRI in the outer membrane was studied using the Fluorokine™ assay (R&D Systems) and a flow cytometer. A similar number of the IPTG-induced cells bound IL-1β compared to non-induced cells (A). Neither group of cells showed significant binding of the control soybean trypsin inhibitor (STI) protein (A). However, the IPTG-induced recombinant *E. coli* cells bound IL-1β more efficiently than the non-induced cells, with the former showing a higher mean fluorescence intensity per positively stained cell than latter (B). When BilRI was expressed in *E. coli* without its signal sequence and purified from the cytoplasm, the obtained protein bound more efficiently to IL-1β than to BSA in a microplate assay (C). However, BilRI bound to IL-1β as efficiently as to STI (C). The negative outer membrane control protein from *A. actinomycetemcomitans* (the N-terminal portion of RcpA [32]) did not bind to IL-1β in the microplate assay (C). N=5 (A and B), and N=3-8 (C). Statistically significant differences are indicated as follows: * p≤0.05, ** p<0.01, *** p<0.001 (Paired T-test).

The *E. coli* cells producing the recombinant full-length BilRI protein bound as much IL-1β as the control cells in which BilRI production was not induced based on estimation of the amount of binding as the number of positively stained cells (Figure 5A). However, when the mean fluorescence intensity was measured in the positively stained cells, it was observed that the recombinant BilRI-producing *E. coli* cells bound IL-1β more efficiently per cell compared to the control cells (Figure 5B). Neither the BilRI-producing *E. coli* cells nor the control cells showed any significant binding of the control protein STI (Figure 5A).

### The soluble recombinant form of BilRI bound IL-1β

The recombinant form of BilRI without the signal sequence, expressed and purified from *E. coli* cytoplasm as a soluble protein, was observed to bind IL-1β in a microplate assay (Figure 5C). Moreover, the level of binding to IL-1β was greater than to the blocking protein BSA (Figure 5C). However, no significant difference was found between IL-1β binding and STI binding (5C). Finally, IL-1β-coated wells bound the negative control protein from *A. actinomycetemcomitans* (N-terminal portion of RcpA) more weakly than BilRI (Figure 5C).

### Different clinical isolates of *A. actinomycetemcomitans* expressed BilRI in the outer membrane facing the extracellular space

**Figure 6:**
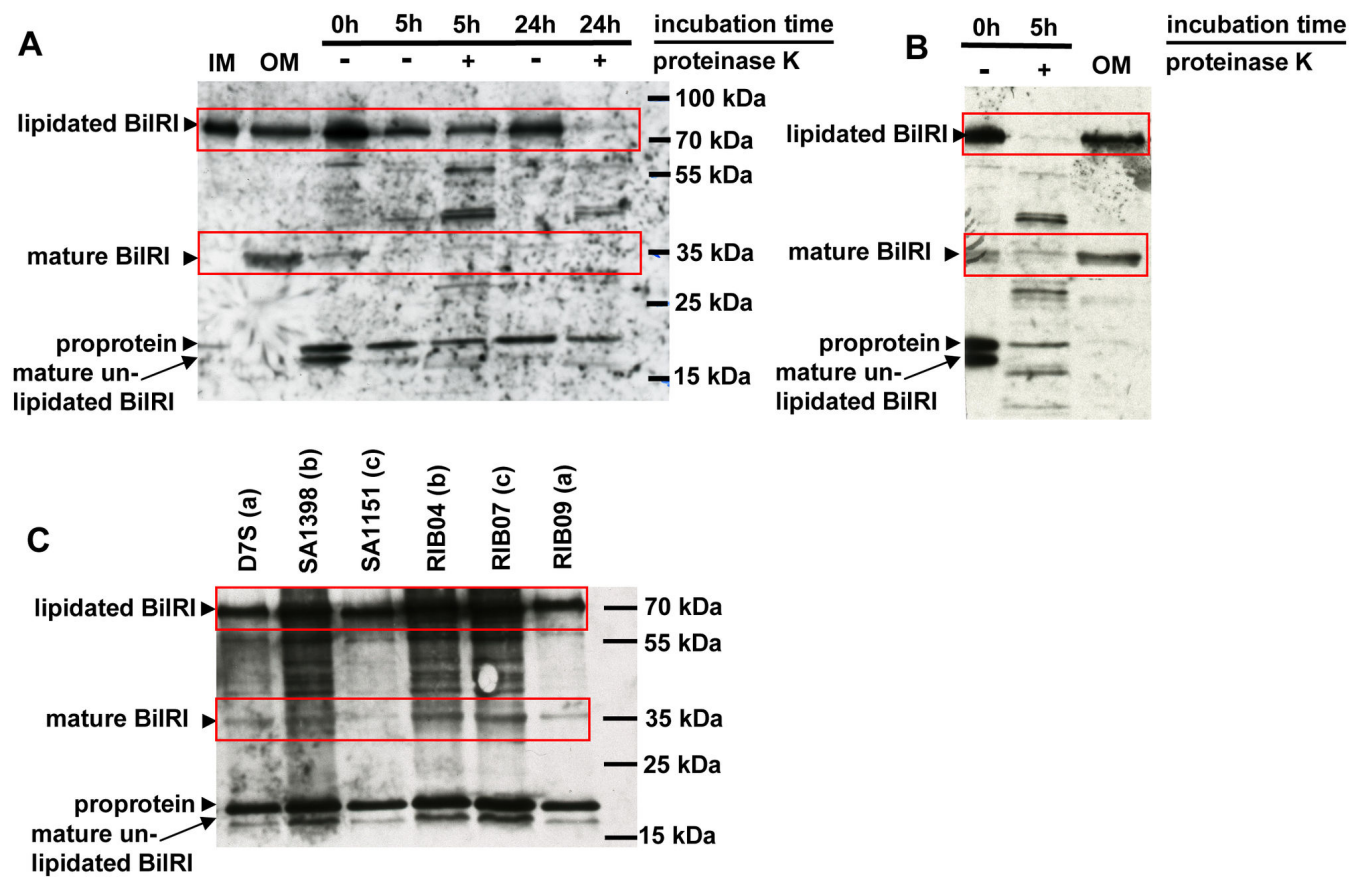
Surface-exposed BilRI was expressed in various clinical isolates of *A. actinomycetemcomitans* grown in biofilms. The surface exposure of BilRI in *A. actinomycetemcomitans* was examined via proteinase K treatment. During proteinase K treatment, protein expression was inhibited with chloramphenicol, and the maturation of the proprotein was inhibited with globomycin. Intact *A. actinomycetemcomitans* D7S cells were incubated with proteinase K for various time periods ranging from 0 to 24 hours, and the cells were then lysed via sonication. The samples were subsequently subjected to SDS-PAGE and immunoblotted with an anti-BilRI antibody. Proteinase K treatment decreased the amount of both forms of BilRI (red quadrangles) detected in outer membrane (OM) protein samples with two different antibody clones, 16B8 (A) and 16F7 (B), suggesting that BilRI was surface exposed in *A. actinomycetemcomitans*. To examine BilRI expression in the clinical isolates, cell lysates were obtained from young (21 hours) *A. actinomycetemcomitans* biofilm cultures through sonication. Samples containing approximately 0.3×10^7^ disrupted cells were subjected to SDS-PAGE and immunoblotted using an anti-BilRI antibody (B). The letters in parenthesis following the strain code indicate the serotypes (B).

The mature BilRI protein traveled to an extracellular position in *A. actinomycetemcomitans* D7S, as proteinase K treatment of whole cells digested BilRI (Figure 6A). The outer membrane protein fraction of strain D7S contained two forms of BilRI, with sizes of approximately 35 kDa and 70 kDa (Figure 6A). The larger of the two was also found in the inner membrane preparate (Figure 6A). We hypothesized that the larger form was a lipidated immature form of BilRI, as large amounts of the smaller, 35 kDa form were only detected in the outer membrane protein fraction (Figure 6A). However, both of these forms are likely surface exposed in the outer membrane, as their respective bands could be eliminated by proteinase K treatment (Figure 6A). These bands could also be detected by another antibody clone, 16F7, in the same samples (Figure 6B). All four forms of BilRI (the proprotein, the unlipidated mature form and the 70 kDa and 35 kDa forms) could be detected in *in vitro* biofilm cultures of all of the tested clinical isolates of *A. actinomycetemcomitans*, which were obtained from periodontitis patients (Figure 6C).

## Discussion

The purpose of this study was to identify an IL-1β-binding receptor in the outer membrane of *A. actinomycetemcomitans*. Various bacterial species are known to display specific receptors for host cytokines [27,29,38], and two of such receptors have been identified [33,34]. Although we were able to demonstrate active binding and internalization of IL-1β in the cells of our model bacterium (*A. actinomycetemcomitans*) and to identify potential intracellular bacterial proteins that might interact with IL-1β in earlier works [31,32], we have not previously been able to identify the bacterial protein that interacts with IL-1β in the outer membrane of the bacterium. Because IL-1β sensing likely leads to the formation of a robust biofilm, discovering the outer membrane IL-1β receptor is crucial for future studies addressing the role of this sensory cascade in the virulence of this opportunistic pathogen.

We discovered that a previously uncharacterized outer membrane lipoprotein of *A. actinomycetemcomitans* bound IL-1β. Recombinant *E. coli* cells which overexpressed the mature protein bound more efficiently IL-1β than the control cells where the recombinant protein expression was not induced. However, the finding that *E. coli* cells had intrinsic ability to bind IL-1β was expected, since *E. coli* has been shown to specifically bind IL-1β [27]. Although the purified recombinant unlipidated form of the protein bound also the control protein STI in ELISA assays, STI did not bind significantly to the whole *E. coli* cells. This might be an indication that a membrane environment is needed for the protein to adapt to a form that binds specifically to IL-1β. The IL-1β binding protein was designated bacterial interleukin receptor I (BilRI) because this is the first identified bacterial IL-binding protein with no other known function than the binding of IL-1β. The first bacterial IL-1β-binding protein to be described was the Caf1A usher protein of *Yersinia pestis* [33], but its main function is to take part to the production and construction of the F1 capsule [39]. An outer membrane protein A (OmpA) ortholog that senses IFN-γ and regulates virulence gene expression through quorum-sensing signaling, OprF of *Pseudomonas aeruginosa* [34], is also involved in the adhesion of *P. aeruginosa* to various host cells [40,41]. The Caf1A usher protein and OprF are both porin proteins [42,43] that form a channel through the outer membrane of Gram-negative bacteria. Bacterial lipoproteins linked to virulence traits can possess various functions, ranging from potential antigen activity to adhesion to host cells, and may play a role in antibiotic resistance (reviewed in 44). Additionally, some bacterial lipoproteins function as receptors or components of more complex transport systems [44]. A recent study showed that the conserved lipoprotein Lpp found in Gram-negative species binds specifically to various cationic α-helical antimicrobial peptides and participates in the internalization of these antimicrobial peptides into the cytoplasm of the bacterium [45]. This demonstrates that bacterial lipoproteins may interact with components of the host innate defense system. Whether BilRI binds only IL-1β, or could it also interact with other host cytokines and chemokines, needs to be confirmed in further studies.

The results of the present study suggest that BilRI is a surface-exposed outer membrane lipoprotein that most likely attaches to the lipid bilayer through its lipid portion. This assumption is supported by the findings that most of the protein forms that were dominant in the outer membrane protein fraction could be digested by proteinase K treatment of whole *A. actinomycetemcomitans* cells and that the protein was highly soluble when produced as a cytosolic form without the signal sequence. The two forms that dominated the outer membrane protein fraction were approximately 35 kDa and 70 kDa in size, which is larger than both the predicted proprotein (19 kDa) and mature unlipidated BilRI (17 kDa). The 70 kDa protein was also present in the inner membrane protein fraction. Thus, we hypothesized that the 35 kDa form corresponded to mature lipidated BilRI, since it was only detected from the whole cells and the outer membrane fraction and not from the inner membrane faction. The level of the 19 kDa proprotein form remained constant throughout proteinase K treatment, as chloramphenicol inhibited protein synthesis, and globomycin impeded the processing of the pro-form to the unlipidated mature form. However, the lipidation and maturation process needs to be studied in more detail to confirm the exact composition of each protein form. It was unexpected to find that the recombinant BilRI attached to the extracellular side of the outer membrane in *E. coli*, as all of the lipoproteins previously identified in *E. coli* travel to the periplasmic side [46]. In summary, BilRI might be among the first proteins that interact with IL-1β during one of the most complex pathways through the cell wall of *A. actinomycetemcomitans*.

Sequence similarity searches revealed that protein sequences similar to BilRI were mainly found in the *Pasteurellaceae* family, including different strains of *A. actinomycetemcomitans* and species such as *Heamophilus* *influenzae* , *Haemophilus parainfluenzae*, *Haemophilus haemolyticus,* *Haemophilus* *somnus* *,* *Aggregatibacter* *aphrophilus* , *Aggregatibacter* *segnis* and *Pasteurella multocida*. However, the function of the protein is unknown in majority of these strains. The only known function among these homologous proteins has been identified for *Haemophilus* *ducrey* fibrinogen binder A (FgbA), which binds to human fibrinogen and is an important virulence factor in humans [47]. However, we cannot claim that a similar protein cannot be found in other bacterial families, as similar binding structures can be formed from different amino acid sequences. Therefore, it is important to also determine the three-dimensional structure of BilRI to find similar receptors in other species.

Overexpression of BilRI in the outer membrane of *E. coli* made the cells brittle and prone to lysis when they were frozen without a supplementary protective agent, such as glycerol. This finding was unexpected because we used an *E. coli* strain that was specifically designed for the production of outer membrane proteins. However, the vulnerability of the outer membrane of Gram-negative bacteria may restrain the recombinant production of outer membrane proteins containing an appropriate signal sequence, which has complicated the use of Gram-negative species as host strains in cell surface display applications [48]. We attempted to overcome this problem by shortening the IPTG induction time from three to two hours, in addition to freezing the cells in the presence of 50% glycerol. Further research is needed to decipher how the overexpression of BilRI affects the membrane integrity of *A. actinomycetemcomitans*.

In conclusion, we identified a potential first-line binder and receptor for the central human proinflammatory cytokine IL-1β from the opportunistic periodontal pathogen *A. actinomycetemcomitans*. The interest in this finding is increased by the fact that no other functions have been described for this protein or its homologs in other bacterial species. Although specific binding of IL-1β by various Gram-negative and Gram-positive bacterial species has been reported [27,29,33], the identified BilRI protein could only be found from the family *Pasteurellaceae*. Future studies addressing the three-dimensional structure of the receptor will resolve whether the protein structure is entirely novel, or if it shared with other proteins present in different species. Since human mononuclear leukocytes detect *A. actinomycetemcomitans* as a pathogen leading to the production of biologically active IL-1β [25], the findings reported here present new opportunities for studying the role of IL-1β uptake in the virulence of *A. actinomycetemcomitans* and in host–pathogen crosstalk.

## Materials and Methods

### Ethics Statement

Permission to collect and use clinical bacterial strains from *A. actinomycetemcomitans* positive patients was obtained from the Ethics committee of the Hospital District of Southwest Finland, Turku, Finland. Subgingival microbial samples from adult periodontitis patients were obtained with written informed consent.

### Identification of an IL-1β-binding membrane protein

The total membrane protein fraction was isolated from *A. actinomycetemcomitans* D7S [49] using a previously described protocol [50] with some modification. Briefly, plate-grown (tryptic soy agar (TSA), 5% defibrinated sheep blood) *A. actinomycetemcomitans* cells (8 plates/extraction) were suspended in PBS_1_, pH 7.4 (10 mM Na_2_HPO_4_, 1.8 mM KH_2_PO_4_, 140 mM NaCl, 2.7 mM KCl), in a total volume of 30 ml. The cells were then centrifuged (4,000×g, 20 min, 4^°^C) and resuspended in 8 ml of PBS_1_-saccharose (PBS_1_, 150 mM saccharose, 1 mM Pefablock SC [Roche Diagnostics, Indianapolis, IN, USA]), disrupted via sonication (4×1 min on ice, separated by 1 min cooling periods), and whole cells and cell debris were removed via centrifugation (1 700×g, 20 min, 4^°^C). The supernatant was subsequently divided into two 6.5 ml ultracentrifugation tubes (#355645, Beckman Instruments Inc., Palo Alto, CA, USA), and 500 µl of a 900 mM saccharose solution in PBS_1_ was pipetted below the supernatant using a capillary pipette. The tubes were then centrifuged (150,000×g, 2 hours 45 min, 4^°^C) to separate the membrane proteins from the soluble ones. The membrane proteins were located in the pellets, which were stored at -20^°^C for further use following removal of the supernatant.

One membrane protein pellet was dispersed in 400 µl of PBS_1_ containing 4% CHAPS (Sigma) and 1 mM phenylmethylsulfonyl fluoride (PMSF; Sigma), first by careful pipetting and then through slow rotation for 30 min at RT and 2.5 hours at 37^°^C. The remaining insoluble material was removed by spinning briefly in a minifuge. The protein content in the supernatant was determined using the method described by Lowry et al. [51]. Membrane proteins (total amount of 2 µg) had been solubilized as described above were incubated with 300 ng of recombinant IL-1β (ReliaTech, GmbH, Braunschweig, Germany) for 1 hour at RT, after which the samples were run in a non-denaturing 4-15% Tris-HCl precast gel (Criterion, Bio-Rad, USA) and transferred to nitrocellulose membranes (Protran®Whatman®, Dassel, Germany) in an Amersham Biosciences Semi-dry blotter. Two controls were included in each gel, one of which contained only the membrane proteins, while the other contained only IL-1β. The membranes were blocked with 5% skimmed milk in PBS_1_ containing 0.05% Tween-20 (PBS_1_-T) at RT for 1 hour, washed twice with PBS_1_-T at RT for 10 min and incubated with a rabbit anti-IL-1β antibody (NB600-633; Novus 280 Biologicals, Littleton, CO), diluted 1:4,000 in PBS_1_-T containing 0.5% skimmed milk at 4^°^C overnight. Following incubation with the primary antibody, the membrane was washed four times with PBS_1_-T for 5 min each and incubated with IRDye® 800CW Donkey Anti-Rabbit IgG (H+L) (#926-32213, LI-COR Biosciences, Lincoln, NE, USA), diluted 1:10,000 in PBS_1_-T, at RT for 1 hour. Prior to detection with Odyssey Infrared Imaging System (LI-COR Biosciences), the membrane was washed six times with PBS_1_-T for 5 min and then twice with PBS_1_ for 5 min. Identical sample series were run and visualized using silver staining [52].

A silver-stained protein band that reacted with IL-1β was cut from a lane containing both MP and IL-1β. The protein sample that was cut from the gel was in-gel digested with trypsin, and the resultant peptides were analyzed with a nanoflow HPLC system (EasyNano, Thermo, Fisher Scientific, Bremen, Germany) coupled to an LTQ Orbitrap Velos Pro mass spectrometer (Thermo, Fisher Scientific) equipped with a nano-electrospray ionization source. The peptides were first loaded onto a trapping column and were subsequently separated inline on a 15 cm C18 column (75 µm × 15 cm, Magic 5 µm 200 Å C_18_, Microm BioResources Inc., Sacramento, CA, USA). MS data were acquired automatically using Thermo Xcalibur software (Thermo, Fisher Scientific). An information-dependent acquisition method was employed, which consisted of a TOF MS survey scan with a mass range of 300-2,000 m/z. The ten most intensive peaks were selected for fragmentation. The obtained data files were searched for protein identification using Proteome Discover (1.3) connected to in-house Mascot (v 2.4) software against the UniProt database (release 2012_06). Only proteins with at least one “bold red” peptide were included in further analyses, as “bold red” indicated a peptide that was a best match for the assigned protein. Protein hits against species other than *A. actinomycetemcomitans* or humans were filtered out. Protein hits of less than two peptides were also removed.

### Bioinformatics

The SOSUI-GramN server [53] was employed to predict the sub-cellular location of the protein. The sequence was analyzed using the SignalP 4.1 Server [54] to predict the length of the signal sequence and the LipoP 1.0 Server [55] to detect the presence of the lipoprotein signal sequence. Sequence similarity searches were performed with SIB using the BLAST network service. The SIB BLAST network service employs a server developed at SIB and NCBI BLAST 2 software [56]. Sequence alignments were performed using the Clustal W (1.83) program of the SIB T-Coffee multiple sequence alignment package [57].

### Cloning and expression of the IL-1β-binding membrane protein

The cloning of *bilRI* was performed as described for other recombinant *A. actinomycetemcomitans* proteins [32], with slight modifications. NdeI and XhoI restriction sites were introduced into the forward and reverse primers, respectively, and are underlined in the primers. The forward primer for *bilRI* was 5’-ATAC A T A T GAAAAAATCAGTATTAGCC-3’, and the reverse primer was 5’-ATAC T C G A GTTATTTGCTTTCAGTTTC-3’ (Eurofins MWG Operon, Ebersburg, Germany). The Phusion™ High-Fidelity DNA polymerase (Finnzymes, Espoo, Finland) was used to amplify the *bilRI* gene from *A. actinomycetemcomitans* D7S DNA. An annealing temperature of 54^°^C was selected for the amplification reaction. The obtained PCR products were digested with NdeI and XhoI (Fermentas, Sankt Leon-Rot, Germany) and cloned into the pET36b vector (Novagen, Darmstadt, Germany) using T4 Ligase (Fermentas). The plasmids were then transformed into *E. coli* TOP10 cells (Invitrogen) via electroporation. Potential plasmid constructs were sequenced in both directions using Eurofins MWG Operon.

A plasmid construct that was verified to contain the correct *bilRI* sequence was transformed into the *E. coli* C41(DE3) RIL strain (Lucigen, Middleton, WI, USA), which was designed specifically for the production of membrane proteins [36]. Various growth temperatures (room temperature [RT], 30^°^C, 37^°^C), isopropyl β-D-thiogalactoside (IPTG) concentrations (0.1, 0.5, 1.0 mM) and induction times (3 hours, overnight) were tested to find the optimal conditions for the production of the outer membrane lipoprotein. Outer membrane proteins were extracted from *E. coli* [58] and analyzed via SDS-PAGE with silver staining [52]. The recombinant protein was identified from a silver-stained gel via mass spectrometry, as described above. Based on the results of these analyses, the expression of BilRI was induced for 3 hours with 0.1 mM IPTG when cells first reached an optical density of 0.6 at 600 nm in a special medium (10 g/l tryptone, 24 g/l yeast extract, 2.5 g/l KCl, 2.5 g/l NaCl, 0.6 g/l NaOH) containing 30 µg/ml kanamycin and chloramphenicol at 37^°^C.

### Determining the location of BilRI expressed in *E. coli*

The cellular fractions (cytosolic, inner membrane, outer membrane) were extracted as described above and the presence of recombinant BilRI was detected using silver staining. The purity of the outer membrane fraction was confirmed by the absence of inner membrane located heme proteins [37]. The samples of different fractions, i.e. soluble cytosolic, sarkocyl soluble inner membrane, and outer membrane dissolved in 2% SDS, were dot blotted in nitrocellulose membrane and the heme proteins were detected with ECL (Pierce®, Thermo Life science) [59].

### Effect of overexpressed outer membrane BilRI on *E. coli* membrane frailty and the self-aggregation of the recombinant cells

Recombinant *E. coli* cells containing the complete BilRI sequence were induced with IPTG as described above. Non-induced cells were used as controls. The cells were harvested (2000×g, 10 min, 4^°^C) and washed twice with PBS_1_. The cell pellets were frozen to -20^°^C and then resuspended in PBS_1_. Next, the cell suspensions were centrifuged (16,000×g, 15 min, 4^°^C), and 10 µl of the supernatant was run in a 0.8% agarose gel containing the Midori Green DNA Stain (Nippon Genetics Europe, Düren, Germany). To determine the optimal freezing conditions for the recombinant *E. coli* cells, cell pellets were suspended in different concentrations (20-50%) of glycerol before freezing, and cell breakage was studied as described above.

Recombinant *E. coli* cells that had been stored in 50% glycerol were washed twice with HEPES buffer (10 mM HEPES, pH 7.4) and suspended at an OD_600nm_=1 in HEPES buffer supplemented with 50 mM CaCl_2_. *E. coli* cells in which the production of BilRI was not induced were used as controls. The cells were incubated in the CaCl_2_-supplemented buffer for 1 hour at 37^°^C, after which the self-aggregation of the cells was estimated visually.

### Determination of the IL-1β-binding capacity of *E. coli* cells expressing BilRI

BilRI expression was induced in *E. coli* as described above, except that the IPTG induction time was shortened to 2 hours. Cells stored in 50% glycerol were washed three times with PBS_1_ (5,900×g, 10 min, 4^°^C) prior to being fixed in PBS_1_ containing 1% formaldehyde, 1% BSA and 0.01% EDTA for 2 hours at 4^°^C. We applied the same fixation conditions which were in a previous study, and were found to preserve the IL-1β-binding capacity of *A. actinomycetemcomitans* [32]. The number of cells in the *E. coli* samples was adjusted to 10^8^ using the OD-specific cell concentration conversion, according to which an OD_600_=1 for *E. coli* cells cultured in LB medium is equal to 7.8±0.8x10^8^ cell/ml [60]. Following the adjustment of cell numbers, the cells were washed once with PBS_1_, collected via centrifugation and resuspended in 1 ml of PBS_1_. For flow cytometric assays, the reagents from a commercial Fluorokine® kit (NFLB0, R&D Systems) were used for cell staining. First, 2.5x10^6^ cells in a final volume of 25 µl were mixed with 10 µl of biotinylated IL-1β or 10 µl of the biotinylated control protein STI prior to incubation at 4^°^C for 1 hour. Then, 10 µl of the avidin-FITC label, or 5 µl of Syto9 (*LIVE/DEAD* ® Bac*Light™* Bacterial Viability and Counting Kit, L34856), diluted 1:5 in sterile water, was added to the reaction mixture, and incubation was continued at 4^°^C for 30 min. Finally, the avidin-FITC-labeled samples were washed twice with 1x RDF1 buffer prior to resuspension of the cell pellets in 1 ml of the buffer. The samples were analyzed with a Cell Lab Quanta SC flow cytometer (Beckman Coulter, Inc.). During a flow cytometric run, the bacterial cells were excited at 488 nm by an argon ion laser. The green fluorescence of FITC-labeled avidin binding biotinylated IL-1β on the cell surface or Syto9-stained nucleic acids was detected through a 525 nm band pass filter. The fluorescence signals were amplified in logarithmic mode. Two parameters (the mean fluorescence intensity [MFI] and the percentage of fluorescence-positive bacterial cells) were determined separately from approximately 10,000 bacteria at a flow rate of 200–300 events/s by gating the bacterial population according to the green fluorescence/side scatter (SSC) bivariate histogram. To exclude disturbing debris in the green fluorescence/SSC histogram, the discriminant was set to the SSC channel. Additionally, Syto9, which was the dye used for staining nucleic acids in both live and dead bacteria, was used to determine the actual proportion of bacteria in the sample.

### Expression and purification of the cytosolic soluble form of BilRI

According to the obtained amino acid sequence, BilRI was predicted to contain a 19 amino acid-long signal sequence typical of Gram-negative membrane lipoproteins. To produce the soluble cytosolic form of the protein, the gene was cloned without the signal sequence-coding region using the following primers: forward, 5’-ATTCATATGTGTGATGACAGCAAAACTTC-3’; reverse, 5’-ATACTCGAGTTTGCTTTCAGTTTCGC-3’. The gene was then cloned into the pET36b vector. However, during this assessment, the recombinant protein did not contain the translation stop codon, and the 8-histidine coding tag was translated from the plasmid to the C-terminal end of the recombinant protein. PCR amplification and plasmid construction were performed as described above, and the plasmids were electroporated into *E. coli* XL1 blue cells. The potential plasmid constructs were sequenced in both directions using the Eurofins MWG Operon.

The correct plasmid construct was transformed into BL21-CodonPlus (DE3)-RIL cells (Stratagene, La Jolla, CA, USA). The expression of cytosolic BilRI was induced for 3 hours with 0.1 mM IPTG when the cells reached an optical density at 600 nm of 1.3 in TB medium (12 g/l tryptone, 24 g/l yeast extract, 0.4% glycerol, 23.1 g/l KH_2_PO_4_ and 125.4 g/l K _2_HPO_4_) containing 30 µg/ml kanamycin and chloramphenicol at 37^°^C. The cells (10 g) were harvested (5,000×g, 10 min, 4^°^C) and dissolved in Buffer A (50 mM Na-phosphate, 800 mM NaCl, 20 mM imidazole, pH 7.5), to which a small amount of DNase I (Roche Diagnostics, Mannheim, Germany) and 0.2 mM PMSF were added. The cells were disrupted via sonication (4×15 s, separated by 1 min of incubation on ice), and intact cells and cell debris were collected via centrifugation (36,000×g, 30 min, 4^°^C). The supernatant, containing soluble BilRI, was applied to a HisTrap™HP column (Amersham Biosciences), then washed with 10% Buffer B (50 mM Na-phosphate, 800 mM NaCl, 0.5 M imidazole, pH 7.5), and BilRI was eluted with 40% Buffer B. Fractions that contained recombinant BilRI (eluted with 40% and 100% Buffer B) were pooled and purified through size-exclusion chromatography (Superdex 200 26/60 column; GE Healthcare) and equilibrated with PBS_2_ (10 mM Na_2_HPO_4_, 145 mM NaCl, pH 7.2). Finally, the fractions containing BilRI were pooled, concentrated and stored at −70°C prior to use.

### Interaction of the cytosolic soluble form of BilRI with IL-1β

The IL-1β-binding capacity of purified recombinant BilRI was examined using a microplate assay, similar to the method we employed to study the interaction with the DNA-binding protein HU [31], with slight modifications. A total of 100 ng of recombinant IL-1β was bound to each well, and the applied concentration of BilRI was 100 µg/ml. Bound recombinant BilRI was detected with His-Probe™-HRP (Thermo Scientific) and ABTS. As controls, similar amounts of STI (Sigma) and BSA (Sigma) were immobilized. The recombinant N-terminal portion of the outer membrane RcpA protein of *A. actinomycetemcomitans* [32] was used as a negative control protein that did not show significant binding to IL-1β.

### Selection and screening of BilRI-recognizing antibody fragments

The synthetic single-chain antibody fragment (scFv) phage libraries ScFvM and ScFvP were cloned into the pEB32x phagemid. The methods used for M13 phage display, the cloning of the scFvs into the screening vector and the expression of scFv-AP (AP = bacterial alkaline phosphatase) fusion proteins were described by Huovinen et al. [61]. The ScFvP library was originally reported by Brockmann et al. [62]. Briefly, purified recombinant soluble BilRI was immobilized on Dynabeads® M-270 Epoxy (Life Technologies Inc.) using 0.3 mg of antigen per mg of beads, according to the instructions of the Dynabeads® Antibody Coupling Kit. The two antibody phage libraries were mixed in a 1:1 ratio for selection. The total phage input was 5x10^12^ colony-forming units in the first round and 5x10^10^ in the second round. The mass of antigen-coupled beads used in the selections was 1 mg or 0.1 mg, respectively. The phage were incubated with the beads in TBS_1_ (50 mM Tris-HCl, 150 mM NaCl, pH 7.5) containing 0.05% Tween-20 and either 1% milk (1^st^ round) or 1% BSA (2^nd^ round) for 1 hour at RT with rotation. The unbound phage were removed by washing two (1^st^ round) or three times (2^nd^ round) with the buffer used during binding, followed by one wash with TBS_1_ + 0.05% Tween-20 and one wash with TBS_1_. Elution of the bound phage was performed with trypsin.

For single-clone immunoactivity screening, scFvs were cloned from the phagemid (second-round output) into the pLK06H vector using the *Sfi*I restriction enzyme and expressed as scFv-AP fusion proteins in XL1-Blue (Stratagene) in a 96-well format. To test the activity of antibody fragments in sandwich immunoassays, BilRI was immobilized on Maxisorb plates (Nunc A/S, Thermo Fisher Scientific) (100 ng/100 µl/well in PBS_3_, pH 7.4 [10 mM Na_2_HPO_4_, 2 mM KH_2_PO_4_, 37 mM NaCl, 2.7 mM KCl]) through incubation at 4°C, overnight. After removing the unbound antigen, the wells were blocked with TBS_1_ + 1% milk for 2 hours, followed by the addition of the scFv-AP sample (bacterial cell lysate). pNPP (1 mg/ml 4-nitrophenyl phosphate disodium salt hexahydrate [Sigma-Aldrich, UK] in 500 mM Tris-HCl, 200 mM NaCl, 10 mM MgCl_2_, pH 9.0) was used as a substrate for detection. Color development was measured with a Victor Multilabel counter (PerkinElmer/Wallac, Finland) at 405 nm. Ten active clones were identified and produced in 50 ml volumes, then extracted from the cells using the freeze-thaw method. A lysate was employed in the experiments after removing cell debris via centrifugation. The capability of the active clones to bind BilRI was verified through western blotting, and clone 16B8 was selected for use in further analyses.

### Expression of BilRI in various clinical isolates of *A. actinomycetemcomitans*

Clinical isolates of *A. actinomycetemcomitans* were collected from periodontitis patients. Subgingival microbial samples from adult periodontitis patients were obtained, with written informed consent, at the Community Dental Health Care Center of Turku (Institute of Dentistry, University of Turku) by students or dentists as part of periodontal examinations and treatment. The samples were collected at baseline or at the treatment evaluation appointment, if disease still existed. *A. actinomycetemcomitans* was detected in the samples via either PCR or culturing. Both chronic and aggressive periodontitis patients were included in the study. Patients were excluded if they had been treated with antibiotics during the past three months, were pregnant, had severe health problems or were on immunosuppressive medications. Patient smoking was recorded. *A. actinomycetemcomitans* strains were cultured at the Helsinki University Hospital Laboratory (HUSLAB, Helsinki, Finland), and the strains were further PCR serotyped [63,64] at the Department of Biochemistry and Food Chemistry, University of Turku. Additionally, three clinical strains, D7S (serotype a), SA1398 (serotype b) and SA1151 (serotype c), were used, which have shown IL-1β-binding capacity in our earlier studies [32]. Biofilm cultures were generated as we described for previous IL-1β binding assays [32]. Briefly, biofilms were cultured in cell culture bottles using a total culture volume of 5 ml and an inoculum of 5×10^8^ cells. The biofilms were first cultured in TSB medium supplemented with 0.6% yeast extract and 0.8% glucose in a candle jar at 37^°^C for approximately 18 hours, after which they were washed twice with 10 ml of PBS_1_. Biofilm growth was continued in RPMI-1640 medium supplemented with 4.1 mM glutamine (Sigma) for another 3 hours, prior to the collection of the biofilm cells in 1 ml of PBS_1_ with a cell scraper. The cells were suspended in Laemmli SDS-PAGE sample buffer at a final concentration of 450 mg/ml, corresponding to approximately 9×10^9^ CFU/ml. Then, the cells were disrupted through sonication on ice (8-12 microns, 4x1 min, one minute break between each sonication), samples were boiled for 5 min, and aliquots containing 0.3×10^7^ disrupted cells were run in 10.5-14% Tris-HCl precast gels (Criterion, Bio-Rad), after which they were transferred to nitrocellulose membranes in an Amersham Biosciences Semi-dry blotter. The membranes were blocked with 2.5% BSA in TBS_2_-T (25 mM Tris, 0.15 M NaCl, 0.05% Tween-20, pH 7.6) at 4^°^C overnight, followed by washing twice with TBS_2_-T for 5 min. The membrane was then incubated with the alkaline phosphatase-fused recombinant anti-BilRI antibody clone 16B8, diluted to 1:500 in 0.5% BSA in TBS_2_-T, at RT for 2 hours and washed again twice as described above. The bound anti-BilRI antibody was detected using a 1:1,000 dilution (in 0.5% BSA supplemented TBS_2_-T) of a biotinylated anti-alkaline phosphatase antibody (NB600-500; Novus Biologicals, Cambridge, UK) at RT for 2 hours, after which the secondary antibody was detected with HRP-labeled streptavidin (S2438, Sigma), diluted to 250 ng/ml in 0.5% BSA in PBS_1_-T, at RT for 2 hours. Finally, the membrane was washed with PBS_1_-T and detected using the ECL substrate (Pierce^®^, Thermo Scientific) and Biomax Light film (Kodak, Rochester, NY, USA).

### Proteinase K treatment to examine the surface exposure of BilRI in *A. actinomycetemcomitans*

To examine the surface exposure of BilRI in *A. actinomycetemcomitans* cells, a slightly modified version of a previously published proteinase K treatment protocol [65] was employed. *A. actinomycetemcomitans* D7S cells that had been cultured for 3 days on TSA plates were suspended in PBS_1_ and collected via centrifugation at 3,800×g. The pellets were resuspended in PBS_1_, and the suspensions were filtered through a 100 mm Nylon Cell Strainer (BD FalconTM #2360). The cell density was adjusted with Proteinase K buffer (50 mM Tris-HCl pH 7.5, 5 mM CaCl_2_, 40 µg/ml chloramphenicol) supplemented with 55 µg/ml globomycin (G1424, Sigma) to 1.7x10^8^ cells/ml. Chloramphenicol was used to hamper protein synthesis, and globomycin was used to inhibit SPII function [66]. Pre-treatment was performed by shaking at 37^°^C for 30 minutes. Then, proteinase K was added to 4.5x10^7^ treated bacteria to a final concentration of 2 mg/ml. In the control samples, proteinase K was replaced with sterile water. The proteolysis reactions were performed through rotation at 37^°^C for 5 hours or 21 hours, before the reactions were stopped by the addition of PMSF at a 1 mM final concentration. The cells were then collected and washed with proteinase K buffer supplemented with 1 mM PMSF. Finally, the pellets were suspended in Laemmli buffer and lysed via sonication. To remove intact cells, the samples were centrifuged at 1,100×g for 20 minutes. The soluble fraction was boiled prior to loading the samples into 10.5-14% Precast Tris-HCl Gels (Criterion, Bio-Rad). Additionally, the inner and outer membrane fractions of *A. actinomycetemcomitans* were extracted as described by Paul-Satyaseela et al. [50] and were used as control samples.

The proteins in the gels were electroblotted onto nitrocellulose membranes. When the proteolysis of BilRI was investigated, the membrane was blocked with 3% BSA in TBS_2_-T at 4^°^C overnight. All remaining steps were performed at RT. First, the membrane was washed twice with TBS_2_-T prior to a 2 hour incubation with a 1:500 dilution of an anti-BilRI alkaline phosphatase-conjugated antibody in TBS_2_-T supplemented with 0.5% BSA (BSA/TBS_2_-T). The primary antibody was custom made through the M13 phage display procedure, as described above. The membrane was subsequently washed twice with TBS_2_-T and incubated with a 1:1,000 dilution of a polyclonal bacterial anti-alkaline phosphatase antibody conjugated to biotin (NB600-500, Novus biologicals) in BSA/TBS_2_-T for 2 hours. Following washing, the membrane was incubated with 250 ng/ml HRP-labeled streptavidin (S2438, Sigma) in BSA/TBS_2_-T for 1 hour and washed several times with TBS_2_-T prior to the addition of the ECL Western blotting substrate (Pierce®, Thermo Scientific). Biomax Light film (Kodak) was used in the detection step. The outer membrane protein RcpA was used as a positive control in proteolysis analysis and was immunostained accordingly. The membrane was blocked with 5% skimmed milk in PBS_1_ supplemented with 0.05% Tween-20 (PBS_1_-T) at RT for 1 hour. After washing twice with PBS_1_-T the membrane was incubated with a rabbit polyclonal anti-RcpA antibody (0.8 µg/ml; Abcell, Tampere) in PBS_1_-T containing 0.5% skimmed milk at 4°C overnight. The next day, the membrane was incubated with an ECL™Rabbit IgG, HRP-linked whole Ab (5.8 ng/ml; NA934, GE Healthcare) at RT for 2 hours, after which detection was carried out described for the BilRI experiments.
